# Novel Image Encryption Scheme Based on Chebyshev Polynomial and Duffing Map

**DOI:** 10.1155/2014/283639

**Published:** 2014-03-26

**Authors:** Borislav Stoyanov, Krasimir Kordov

**Affiliations:** Faculty of Mathematics and Informatics, Konstantin Preslavski University of Shumen, 9712 Shumen, Bulgaria

## Abstract

We present a novel image encryption algorithm using Chebyshev polynomial based on permutation and substitution and Duffing map based on substitution. Comprehensive security analysis has been performed on the designed scheme using key space analysis, visual testing, histogram analysis, information entropy calculation, correlation coefficient analysis, differential analysis, key sensitivity test, and speed test. The study demonstrates that the proposed image encryption algorithm shows advantages of more than 10^113^ key space and desirable level of security based on the good statistical results and theoretical arguments.

## 1. Introduction

In recent years, the dynamical chaotic systems have been commonly used for the design of cryptographic primitives featuring chaotic behaviour and random-like properties. In his seminal work [1], Shannon pointed out the excellent possibilities of the dynamical chaotic maps in the communications. He identified two basic properties that the good data encryption systems should have to prevent (resist) statistical attacks: diffusion and confusion. Diffusion can propagate the change over the whole encrypted data, and confusion can hide the relationship between the original data and the encrypted data. Permutation, which rearranges objects, is the simplest method of diffusion, and substitution, that replaces an object with another one, is the simplest type of confusion. The consistent use of dynamical chaotic system based permutation and substitution methods is in the deep cryptographic fundamental.

The authors of [2] used Chebyshev polynomial to construct secure El Gamal-like and RSA-like algorithms. A new more practical and secure Diffie-Hellman key agreement protocol based on Chebyshev polynomial is presented in [3]. In [4], a stream cipher constructed by Duffing map based message-embedded scheme is proposed. By mixing the Lorenz attractor and Duffing map, a new six-dimensional chaotic cryptographic algorithm with good complex structure is designed [5]. In [6], an improved stochastic middle multibits quantification algorithm based on Chebyshev polynomial is proposed. Three-party key agreement protocols using the enhanced Chebyshev polynomial are proposed in [7, 8].

Fridrich [9] describes how to adapt Baker map, Cat map, and Standard map on a torus or on a rectangle for the purpose of substitution-permutation image encryption. In [10], a new permutation-substitution image encryption scheme using logistic, tent maps, and Tompkins-Paige algorithm is proposed. In [11], chaotic cipher is proposed to encrypt color images through position permutation part and Logistic map based on substitution. Yau et al. [12] proposed an image encryption scheme based on Sprott chaotic circuit. In [13], Fu et al. proposed a digital image encryption method by using Chirikov standard map based permutation and Chebyshev polynomial based diffusion operations.

In [14], a bit-level permutation scheme using chaotic sequence sorting has been proposed for image encryption. The operations are completed by Chebyshev polynomial and Arnold Cat map. An image encryption algorithm in which the key stream is generated by Chebyshev function is presented in [15]. Simulation results are given to confirm the necessary level of security. In [16], a new image encryption scheme, based on Chebyshev polynomial, Sin map, Cubic map, and 2D coupled map lattice, is proposed. The experimental results show the security of the algorithm.

In [17], a color image encryption scheme based on skew tent map and hyper chaotic system of 6th-order CNN is presented. An image encryption scheme based on rotation matrix bit-level permutation and block diffusion is proposed in [18].

A new chaos based image encryption scheme is suggested in this paper. The algorithm is a simple improvement of one round substitution-permutation model. The encryption process is divided in two major parts: Chebyshev polynomial based on permutation and substitution and Duffing map based on substitution. In Section 2, we propose two pseudorandom bit generators (PRBGs): one based on Chebyshev polynomial and the other based on Duffing map. In Section 3, in order to measure randomness of the bit sequence generated by the two pseudorandom schemes, we use NIST, DIEHARD, and ENT statistical packages.  Section 4 presents the proposed image encryption algorithm, and some security cryptanalysis is given. Finally, the last section concludes the paper.

## 2. Proposed Pseudorandom Bit Generators

### 2.1. Pseudorandom Bit Generator Based on the Chebyshev Polynomial

In this section, the real numbers of two Chebyshev polynomials [2, 19] are preprocessed and combined with a simple threshold function to a binary pseudorandom sequence.

The proposed pseudorandom bit generator is based on two Chebyshev polynomials, as described by

(1) $$\begin{array}{c} x\left(n+1\right)=T_{k}\left(x_{n}\right)=\cos\left(k\times \text{arc}\;\cos\left(x_{n}\right)\right),\\ y\left(m+1\right)=T_{l}\left(y_{m}\right)=\cos\left(l\times \text{arc}\;\cos\left(y_{m}\right)\right), \end{array}$$

where (*x*_*n*_, *y*_*m*_)∈[−1,1] and (*k*, *l*)∈[2, *∞*) are control parameters. The initial values *x*(0) and *y*(0) and parameters (*k*, *l*) are used as the key.

*Step  1*. The initial values *x*(0), *y*(0), *k*, and *l* of the two Chebyshev polynomials from (1) are determined.

*Step  2*. The first and the second Chebyshev polynomials from (1) are iterated for *K*_0_ and *L*_0_ times to avoid the harmful effects of transitional procedures, respectively, where *K*_0_ and *L*_0_ are different constants.

*Step  3*. The iteration of (1) continues, and, as a result, two decimal fractions *x*(*n*) and *y*(*m*) are generated.

*Step  4*. These decimal fractions are preprocessed as follows:

(2) $$\begin{array}{c} x\left(n\right)=\mathrm{mod}\;\left(\text{floor}\left(\text{abs}\left(x\left(n\right)\times 10^{14}\right)\right),2\right)\;\\ y\left(m\right)=\mathrm{mod}\;\left(\text{floor}\left(\text{abs}\left(y\left(m\right)\times 10^{14}\right)\right),2\right)\;, \end{array}$$

where abs(*x*) returns the absolute value of *x*, floor(*x*) returns the value of *x* to the nearest integers less than or equal to *x*, and mod⁡(*x*, *y*) returns the reminder after division.

*Step  5*. The following threshold function *g* from (3) is applied:

(3) $$\begin{array}{c} g\left(x\left(n\right),y\left(m\right)\right)=\{\begin{array}{cc} 1, & \text{if}\;x\left(n\right)>y\left(m\right),\\ 0, & \text{if}\;x\left(n\right)\leq y\left(m\right), \end{array}\; \end{array}$$

and a pseudorandom bit is produced. 

*Step  6*. Return to Step 3 until pseudorandom bit stream limit is reached.

### 2.2. Pseudorandom Bit Generator Based on the Duffing Map

In this section, the real numbers of two Duffing maps are preprocessed and combined with a simple threshold function to a binary pseudorandom sequence.

The Duffing map is a 2D discrete dynamical system which takes a point (*u*_*n*_, *v*_*n*_) in the plane and maps it to a new point. The proposed pseudorandom bit generator is based on two Duffing maps, given by the following equations:

(4) u1,n+1=v1,n,v1,n+1=−bu1,n+av1,n−v1,n3,u2,m+1=v2,m,v2,m+1=−bu2,m+av2,m−v2,m3.

The maps depend on the two constants *a* and *b*. These are usually set to *a* = 2.75 and *b* = 0.2 to produce chaotic nature. The initial values *u*_1,0_, *v*_1,0_, *u*_2,0_, and *v*_2,0_ are used as the key.

*Step  1*. The initial values *u*_1,0_, *v*_1,0_, *u*_2,0_, and *v*_2,0_ of the two Duffing maps from (4) are determined. 

*Step  2*. The first and the second Duffing maps from (4) are iterated for *M*_0_ and *N*_0_ times, respectively, to avoid the harmful effects of transitional procedures, where *M*_0_ and *N*_0_ are different constants.

*Step  3*. The iteration of (4) continues, and, as a result, two real fractions *x*(*n*) and *y*(*m*) are generated.

*Step  4*. The following threshold function *h* from (5) is applied:

(5) $$\begin{array}{c} h\left(v_{1,n},v_{2,m}\right)=\{\begin{array}{cc} 1, & \text{if}{\;v}_{1,n}>v_{2,m},\\ 0, & \text{if}\;v_{1,n}\leq v_{2,m}, \end{array}\; \end{array}$$

and a pseudorandom bit is produced. 

*Step  5*. Return to Step* *3 until pseudorandom bit stream limit is reached.

## 3. Statistical Test Analysis of the Proposed Pseudorandom Bit Generators

In order to measure randomness of the zero-one sequence generated by the new pseudorandom generators, we used NIST, DIEHARD, and ENT statistical packages.

The Chebyshev polynomial and the Duffing map based pseudorandom bit schemes are implemented by software simulation in C++ language, using the following initial seeds: *x*(0) = 0.9798292345345, *y*(0) = −0.4032920230495034, *k* = 2.995, *l* = 3.07, *u*_1,0_ = −0.04,  *v*_1,0_ = 0.2,  *u*_2,0_ = 0.23, and *v*_2,0_ = −0.13, stated as a key K1.

### 3.1. NIST Statistical Test Analysis

The NIST statistical test suite (version 2.1.1) is proposed by the National Institute of Standards and Technology [20]. The suite includes 15 tests, which focus on a variety of different types of nonrandomness that could exist in a sequence. These tests are frequency (monobit), block-frequency, cumulative sums, runs, longest run of ones, rank, fast Fourier transform (spectral), nonoverlapping templates, overlapping templates, Maurer's “universal statistical,” approximate entropy, random excursions, random-excursion variant, and serial and linear complexity. The testing process consists of the following steps:

1. state the null hypothesis; assume that the binary sequence is random;
2. compute a sequence test statistic; testing is carried out at the bit level;
3. compute the *P*-value; *P*-value ∈[0,1];
4. fix *α*, where *α* ∈ [0.0001,0.01]; compare the *P*-value to *α*; *Success* is declared whenever *P*-value ≥*α*; otherwise, *failure* is declared.

Given the empirical results from a particular statistical test, the NIST suite computes the proportion of sequences that pass. The range of acceptable proportion is determined using the confidence interval defined as

(6) $$\begin{array}{c} \hat{p}\pm 3\sqrt{\frac{\hat{p}\left(1-\hat{p}\right)}{m}}, \end{array}$$

where $\hat{p}=1-\alpha$ and *m* is the number of binary tested sequences. In our setup, *m* = 1000. Thus the confidence interval is

(7) $$\begin{array}{c} 0.99\pm 3\sqrt{\frac{0.99\left(0.01\right)}{1000}}=0.99\pm 0.0094392. \end{array}$$

The proportion should lie above 0.9805607.

The distribution of *P*-values is examined to ensure uniformity. The interval between 0 and 1 is divided into 10 subintervals. The *P*-values that lie within each subinterval are counted. Uniformity may also be specified through an application of *χ* ^2^ test and the determination of a *P*-values corresponding to the goodness-of-fit distributional test on the *P*-values obtained for an arbitrary statistical test, *P*-values of the *P*-values. This is implemented by calculating

(8) $$\begin{array}{c} \chi^{2}=\sum_{i=1}^{10}\frac{{\left(F_{i}-s/10\right)}^{2}}{s/10}, \end{array}$$

where *F*_*i*_ is the number of *P*-values in subinterval *i* and *s* is the sample size. A *P*-values is calculated such that *P*-value_*T*_ = *IGAMC*(9/2, *χ* ^2^/2), where *IGAMC* is the complemented incomplete gamma statistical function. If *P*-value_*T*_ ≥ 0.0001, then the sequences can be deemed to be uniformly distributed.

Using the proposed pseudorandom Using the proposed pseudorandom bit generators were generated 1000 sequences of 1000000 bits. The results from all statistical tests are given in Table 1.

The entire NIST test is passed successfully: all the *P*-values from all 2 × 1000 sequences are distributed uniformly in the 10 subintervals and the pass rate is also in acceptable range. The minimum pass rate for each statistical test with the exception of the random excursion (variant) test is approximately 980 for a sample size of 1000 binary sequences for both of pseudorandom generators. The minimum pass rate for the random excursion (variant) test is approximately 589 for a sample size of 603 binary sequences for Chebyshev polynomial based PRBG and 604 for a sample size of 618 binary sequences for Duffing map based PRBG. This shows that the generated pseudorandom sequences feature reliable randomness.

Overall, the results confirm that the novel chaotic cryptographic schemes based on Chebyshev polynomial and Duffing map accomplish a very high level of randomness of the bit sequences.

### 3.2. DIEHARD Statistical Test Analysis

The DIEHARD suite [21] consists of a number of different statistical tests: birthday spacings, overlapping 5-permutations, binary rank (31 × 31), binary rank (32 × 32), binary rank (6 × 8), bit stream, Overlapping Pairs Sparse Occupancy, Overlapping Quadruples Sparse Occupancy, DNA, stream count-the-ones, byte count-the-ones, 3D spheres, squeeze, overlapping sums, runs up, runs down, and craps. For the DIEHARD tests, we generated two files with 80 million bits each, from the proposed chaotic pseudorandom bit generators. The results are given in Table 2. All *P*-values are in acceptable range of [0,1). The proposed pseudorandom bit generators passed all the tests of DIEHARD software.

### 3.3. ENT Statistical Test Analysis

The ENT package [22] performs 6 tests (entropy, optimum compression, *χ* ^2^ distribution, arithmetic mean value, Monte Carlo *π* estimation, and serial correlation coefficient) to sequences of bytes stored in files and outputs the results of those tests. We tested output of the two strings of 125000000 bytes of the proposed Chebyshev polynomial based pseudorandom bit generator and Duffing map based pseudorandom bit generator, respectively. The results are summarized in Table 3. The proposed pseudorandom bit generators passed all the tests of ENT.

## 4. Image Encryption Based on Chebyshev polynomial and Duffing Map

Here, we describe an image encryption scheme based on the proposed Chebyshev polynomial and Duffing map based pseudorandom bit generators. We also provide security analysis of the encrypted images.

### 4.1. Encryption Scheme

The proposed image encryption algorithm is modification of the classical substitution-permutation scheme [9], column by column shuffling and masking procedures [23], and the diffusion-substitution model [24]. Here, every single pixel relocation is based on random permutation at once with substitution. The novel derivative scheme has the features of a two-round permutation-substitution color image encryption algorithm. The image encryption method is based on the unique combination of the output bits of the new proposed pseudorandom bit generators.

Without loss of generality, we assume that the dimension of the plain images is *m* × *n* size, where *m* is the number of rows and *n* is the number of the columns. The binary lengths of *m* and *n* are *n*_0_ and *m*_0_, respectively. The encryption process is divided into two stages. In the first stage, we generate buffer image *B* of *m* × *n* size by rearranging and modifying the pixel values of the plain image *P* by Chebyshev polynomial based PRBG. In the second stage, we generate ciphered image *C* of *m* × *n* size by modifying the buffer pixel values by Duffing map based PRBG. The encryption process starts with empty buffer image. The plain image pixels are passed sequentially left to right and top to bottom. The entire encryption process is given below.

*Step  1*. The Chebyshev polynomial based PRBG is iterated continuously to produce *m*_0_ and *n*_0_ bits pseudorandom numbers *i*′ and *j*′ which are transformed modulo *m* and *n*, respectively.

*Step  2*. Repeat Step* *1 until an empty pixel with (*i*′, *j*′) coordinates in the buffer image is detected.

*Step  3*. Continue to do iteration of Chebyshev polynomial based PRBG until 24 bits are produced. 

*Step  4*. To produce buffered image pixel *b*(*i*′, *j*′), do XOR between a plain image pixel *p*(*i*, *j*) and the last generated 24 bits. 

*Step  5*. Repeat Steps* *1–4 until all of the plain image pixels are processed.

*Step  6*. Iterate the Duffing map based PRBG to produce *m* × *n* × 24 bits. Then, do XOR operation between the pseudorandom bit sequence and all of the buffer pixels in the buffered image to produce the encryption image *C*.

For the reasons of security, we propose several overall rounds of the encryption procedure.

### 4.2. Security Analysis

The novel image encryption algorithm is implemented in C++ language. All experimental results discussed in the next subsections have been taken by using one iteration of the scheme.

Sixteen 24-bit color images have been encrypted for the security tests. The images are selected from the USC-SIPI image database, miscellaneous volume, available and maintained by the University of Southern California Signal and Image Processing Institute (http://sipi.usc.edu/database/). The image numbers are from 4.1.01 to 4.1.08, size 256 × 256 pixels, from 4.2.01 to 4.2.07, size 512 × 512 pixels, and House, size 512 × 512 pixels. The chosen images are currently stored in TIFF format and we have converted them into BMP format (24 bits/pixel).

#### 4.2.1. Key Space Analysis

The key space is the set of all possible keys that can be used in encryption/decryption algorithm. The key of the proposed image encryption scheme is that it is produced by the combination of Chebyshev polynomial based PRBG and Duffing map based PRBG. The novel scheme has eight secret keys *x*(0), *y*(0), *k*, *l*, *u*_1,0_, *v*_1,0_, *u*_2,0_, and *v*_2,0_. According to the IEEE floating-point standard [25], the computational precision of the 64-bit double-precision number is about 10^−15^. If we assume the precision of 10^−14^, the secret key's space is more than 10^113^ ≈ 2^375^. This is large enough to defeat brute-force attacks [26] and it is larger than key space size of the image encryption algorithms proposed in [10, 27–29].

Moreover, the initial iteration numbers *K*_0_, *L*_0_, *M*_0_, and *N*_0_ can also be used as a part of the secret key.

#### 4.2.2. Visual Testing

**Figure 1:**
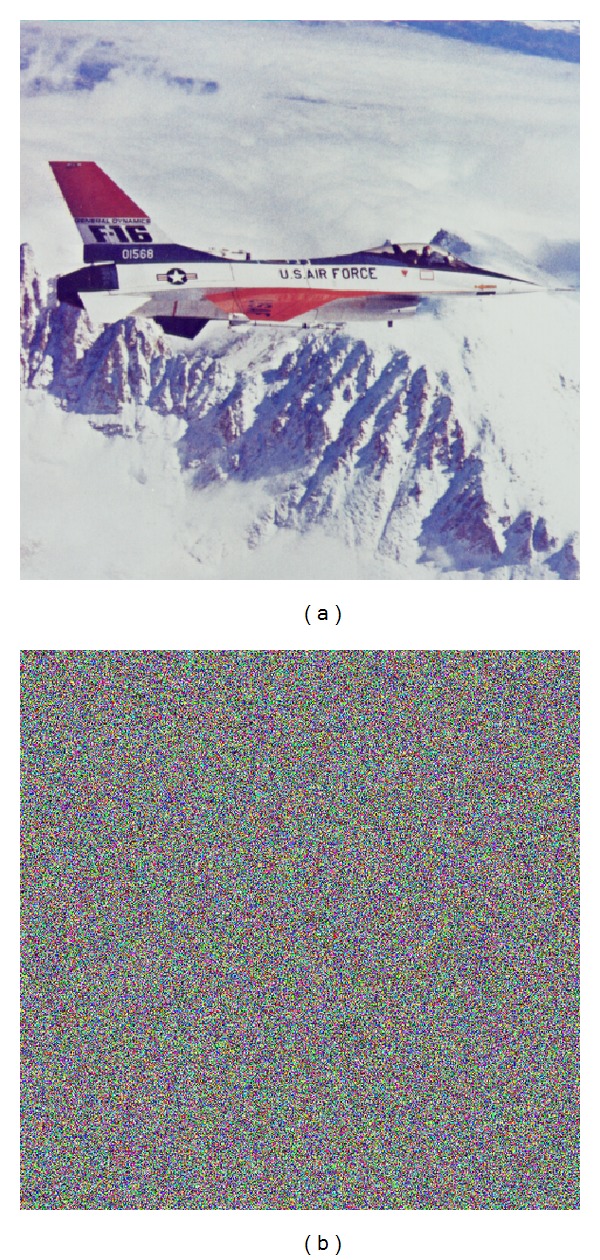
Comparison of the plain image and the encrypted image: (a) original picture 4.2.05 Airplane (F-16); (b) encrypted image of 4.2.05 Airplane (F-16).

The new algorithm is tested using simple visual inspection of the plain images and corresponding encrypted images. The visual observation does not find convergences between every plain image and its encrypted versions. As an example, Figure 1 shows the plain image 4.2.05 Airplane (F-16), Figure 1(a), and its encrypted version, Figure 1(b). The encrypted image does not contain any constant regions in representative color or texture. The proposed chaos based image encryption breaks any visual data from the plain images.

#### 4.2.3. Histogram Analysis

**Figure 2:**
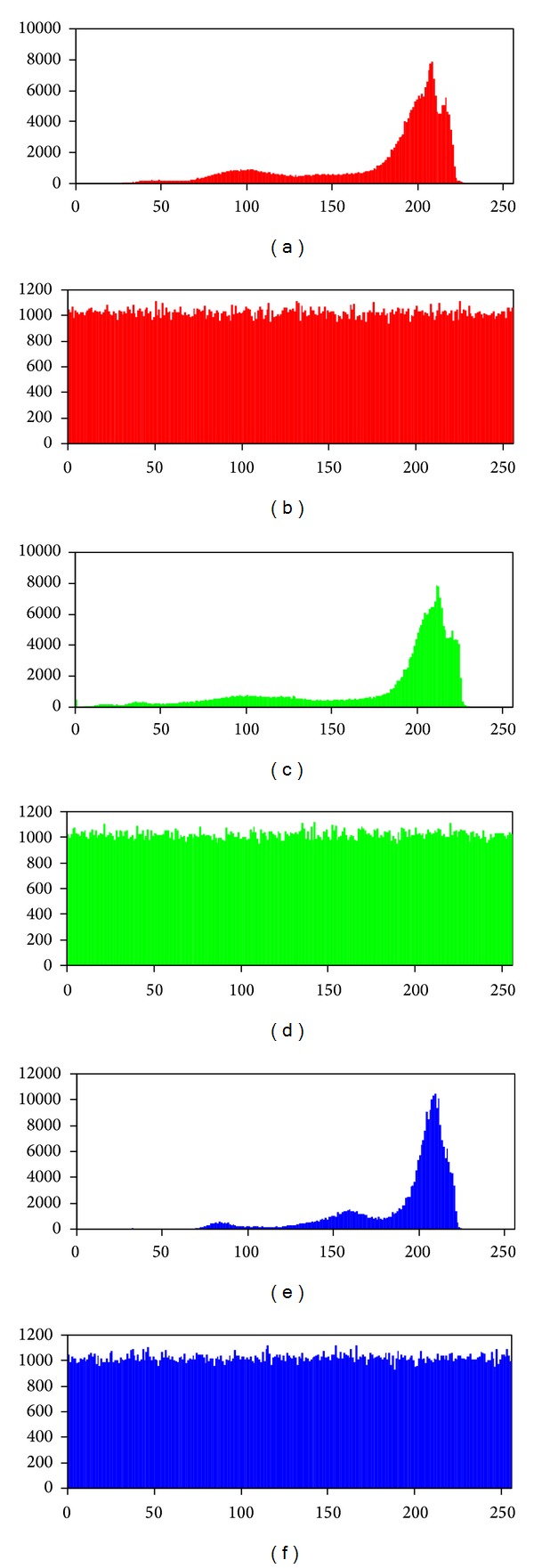
Histogram analysis of plain image and encrypted image: (a), (c), and (e) show the histograms of red, green, and blue channels of plain picture 4.2.05 Airplane (F-16); (b), (d), and (f) show the histograms of red, green, and blue channels of encrypted picture 4.2.05 Airplane (F-16).

An image histogram of pixels is a type of a bar graph. It illustrates the visual impact of a distribution of colors that are at certain intensity. We have calculated histograms of red, green, and blue channels of both plain images and their encrypted version by the new image encryption scheme. One representative example among them is shown in Figure 2. The histograms of encrypted image are completely uniformly distributed and considerably different from that of the plain image.

In addition, the average pixel intensity calculations in Table 4, for all of the encrypted images, confirmed the uniformity in distribution of red, green, and blue channels.

#### 4.2.4. Information Entropy

The entropy *H*(*X*) is statistical measure of uncertainty in information theory [1]. It is defined as follows:

(9) $$\begin{array}{c} H\left(X\right)=-\sum_{i=0}^{255}p\left(x_{i}\right)\text{lo}{\text{g}}_{2}p\left(x_{i}\right), \end{array}$$

where *X* is a random variable and *p*(*x*_*i*_) is the probability mass function of the occurrence of the symbol *x*_*i*_. Let us consider that there are 256 states of the information source in red, green, and blue colors of the image with the same probability. We can get the ideal *H*(*X*) = 8, corresponding to a truly random source.

The information entropy of red, green, and blue channels of the plain images and their corresponding encrypted images are computed and displayed in Table 5. From the obtained values, it is clear that the entropies of red, green, and blue colors of the encrypted images are very close to the best possible theoretical value, which is an indication that the new chaos based image encryption scheme is trustworthy and secure upon information entropy attack.

#### 4.2.5. Correlation Coefficient Analysis

The adjacent pixels in plain images are strongly correlated in either horizontal, vertical, or diagonal direction. The correlation coefficient *r* between two adjacent pixels (*a*_*i*_, *b*_*i*_) is computed in accordance with the way described in [30]. Consider

(10) $$\begin{array}{c} r=\frac{\mathrm{cov}\left(a,b\right)}{\sqrt{D\left(a\right)\sqrt{D\left(b\right)}}}, \end{array}$$

where

(11) $$\begin{array}{c} D\left(a\right)=\frac{1}{M}\sum_{i=1}^{M}{\left(a_{i}-\bar{a}\right)}^{2},\\ D\left(b\right)=\frac{1}{M}\sum_{i=1}^{M}{\left(b_{i}-\bar{b}\right)}^{2},\\ \mathrm{cov}\left(a,b\right)=\sum_{i=1}^{M}\left(a_{i}-\bar{a}\right)\left(b_{i}-\bar{b}\right), \end{array}$$

*M* is the total number of couples (*a*_*i*_, *b*_*i*_), obtained from the image, and $\bar{a}$, $\bar{b}$ are the mean values of *a*_*i*_ and *b*_*i*_, respectively. Correlation coefficient can range in the interval [−1.00; +1.00].

Table 6 shows the results of horizontal, vertical, and diagonal adjacent pixels correlation coefficients calculations of the plain images and the corresponding encrypted images. It is clear that the novel image encryption scheme does not retain any linear dependencies between observed pixels in all three directions: the inspected horizontal, vertical, and diagonal correlation coefficients of the encrypted images are very close to zero. Overall, the correlation coefficients of the proposed algorithm are similar to results of four other image encryption schemes [27–30].

#### 4.2.6. Differential Analysis

In the main, a typical property of an image encryption scheme is to be sensitive to minor alterations in the plain images. Differential analysis supposes that an enemy is efficient to create small changes in the plain image and inspect the encrypted image. The alteration level can be measured by means of two metrics, namely, the number of pixels change rate (NPCR) and the unified average changing intensity (UACI) [30, 31].

Suppose encrypted images before and after one pixel change in plain image are *C*_1_ and *C*_2_. The NPCR and UACI are defined as follows:

(12) $$\begin{array}{c} \text{NPCR}=\frac{\sum_{i=0}^{W-1}\sum_{j=0}^{H-1}D\left(i,j\right)}{W\times H}\times 100\%,\\ \text{UACI}=\frac{1}{W\times H}\left(\sum_{i=0}^{W-1}\sum_{j=0}^{H-1}\frac{\left|C_{1}\left(i,j\right)-C_{2}\left(i,j\right)\right|}{255}\right)\times 100\%, \end{array}$$

where *D* is a two-dimensional array, having the same size as image *C*_1_ or *C*_2_, and *W* and *H* are the width and height of the image. The array *D*(*i*, *j*) is defined by *C*_1_(*i*, *j*) and *C*_2_(*i*, *j*); if *C*_1_(*i*, *j*) = *C*_2_(*i*, *j*), then *D*(*i*, *j*) = 1; otherwise, *D*(*i*, *j*) = 0. The NPCR and UACI test results from the proposed chaos based algorithm are shown in Table 7.

The obtained NPCR values for the images from 4.1.01 to 4.1.08 are larger than critical values *N*_0.05_* = 99.5693, *N*_0.01_* = 99.5527, and *N*_0.001_* = 99.5341 and, for the images from 4.2.01 to 4.2.07 and House image, are larger than critical values *N*_0.05_* = 99.5893, *N*_0.01_* = 99.5810, and *N*_0.001_* = 99.5717 [31].

The obtained UACI values for the images from 4.1.01 to 4.1.08 are in the intervals from *N*_0.05_^∗−^ = 33.2824 to *N*_0.05_^∗−^ = 33.6447, from *N*_0.01_^∗−^ = 33.2255 to *N*_0.01_^∗−^ = 33.7016, and from *N*_0.001_^∗−^ = 33.1594 to *N*_0.001_^∗−^ = 33.7677. The obtained UACI values for the images from 4.2.01 to 4.2.07 and House image are in the intervals from *N*_0.05_^∗−^ = 33.3730 to *N*_0.05_^∗−^ = 33.5541, from *N*_0.01_^∗−^ = 33.3445 to *N*_0.01_^∗−^ = 33.5826, and from *N*_0.001_^∗−^ = 33.3115 to *N*_0.001_^∗−^ = 33.6156 [31].

The results from NPCR and UACI computations indicate that the new image encryption scheme is highly sensitive with respect to small changes in the plain images and has a strong ability of resisting differential cryptanalysis.

#### 4.2.7. Key Sensitivity Test

Another important component of correlation analysis is the key sensitivity test. A good image encryption algorithm should be sensitive with respect to the secret key, that is, a slight modification of the secret key. We encrypted the 16 images with three similar secret keys: K1, K2 (*x*(0) = 0.9798292345346, *y*(0) = −0.4032920230495034, *k* = 2.995, *l* = 3.07, *u*_1,0_ = −0.04, *v*_1,0_ = 0.2, *u*_2,0_ = 0.23, and *v*_2,0_ = −0.13), and K3 (*x*(0) = 0.9798292345347, *y*(0) = −0.4032920230495034, *k* = 2.995, *l* = 3.07, *u*_1,0_ = −0.04, *v*_1,0_ = 0.2, *u*_2,0_ = 0.23, and *v*_2,0_ = −0.13). The results are shown in Table 8. It is evident that the proposed image encryption is highly key sensitive: the calculated correlation coefficients are very close to 0.00.

**Figure 3:**
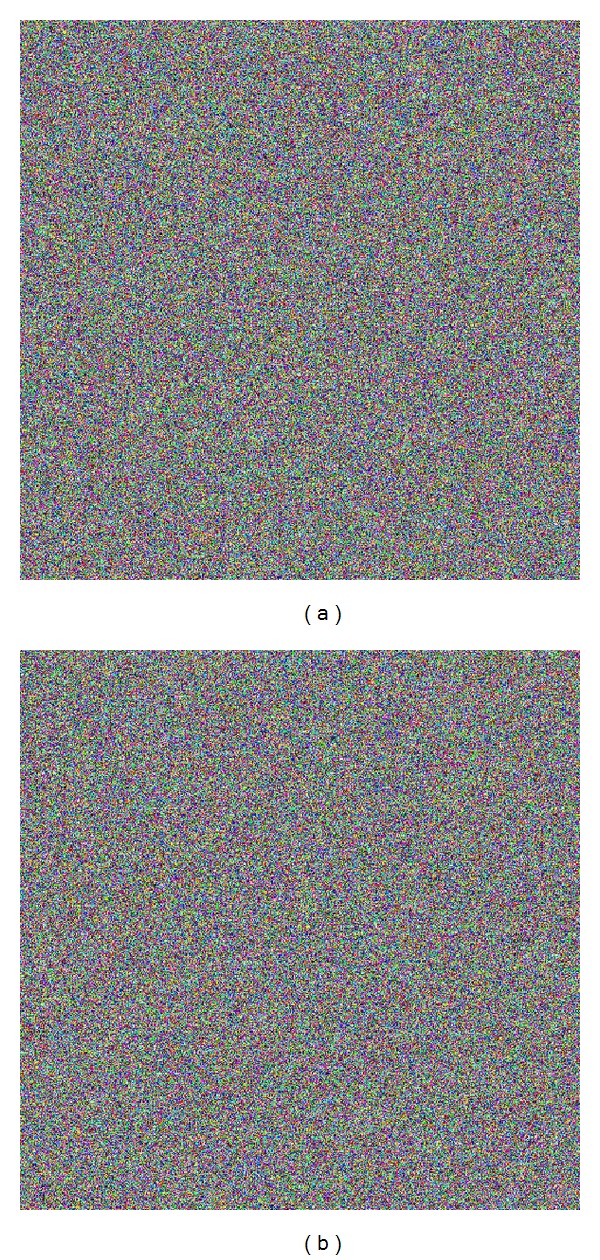
Decryption of Image 4.2.05 Airplane (F-16): (a) encrypted with key K1 and decrypted using key K2 and (b) encrypted with key K1 and decrypted using key K3.

Moreover, in Figure 3, the results of two tests are shown to decrypt the Figure 1(b), with the secret keys K2 and K3.

We observed that the two decrypted images (Figure 3(a) and Figure 3(b)) have no relation with the plain image 4.2.05, Figure 1(a).

#### 4.2.8. Speed Test

We have measured the encryption time for 256 × 256 and 512 × 512 sized images by using the novel image encryption algorithm. Speed analysis has been done on 2.8 GHz Pentium IV personal computer. In Table 9, we compared the speed of our method with [24, 32, 33]. The data show that the proposed image encryption scheme has a satisfactory speed.

## 5. Conclusions

A novel image encryption algorithm based on dynamical chaotic systems is proposed in this paper. The developed encryption scheme combines Chebyshev polynomial based permutation and substitution and Duffing map based substitution. A precise security analysis on the novel encryption algorithm is given. Based on the experimental results of our computations, we conclude that the proposed chaos based image encryption technique is perfectly suitable for the practical image encryption.

## Figures and Tables

**Table 1:** NIST statistical test suite results for 2 × 1000 sequences of size 10^6^-bit each generated by the proposed Chebyshev polynomial based pseudorandom bit generator and Duffing map based pseudorandom bit generator.

NIST statistical test	Chebyshev PRBG		Duffing PRBG	
	*P*-value	Pass rate	*P*-value	Pass rate
Frequency (monobit)	0.649612	990/1000	0.490483	989/1000
Block-frequency	0.455937	991/1000	0.777265	992/1000
Cumulative sums (forward)	0.877083	990/1000	0.660012	988/1000
Cumulative sums (reverse)	0.983938	992/1000	0.284024	987/1000
Runs	0.062427	995/1000	0.490983	993/1000
Longest run of ones	0.215574	993/1000	0.612147	992/1000
Rank	0.848027	991/1000	0.212184	988/1000
FFT	0.194813	993/1000	0.013474	993/1000
Nonoverlapping templates	0.504571	990/1000	0.458442	990/1000
Overlapping templates	0.219006	992/1000	0.279844	988/1000
Universal	0.660012	986/1000	0.278461	991/1000
Approximate entropy	0.000478	990/1000	0.363593	991/1000
Random excursions	0.508738	597/603	0.671829	612/618
Random excursions variant	0.614825	596/603	0.490932	612/618
Serial 1	0.585209	991/1000	0.779188	990/1000
Serial 2	0.767582	989/1000	0.713641	993/1000
Linear complexity	0.711601	986/1000	0.699313	991/1000

**Table 2:** DIEHARD statistical test results for two 80 million bits sequences generated by the proposed Chebyshev polynomial based pseudorandom bit generator and Duffing map based pseudorandom bit generator.

DIEHARD statistical test	Chebyshev PRBG	Duffing PRBG
	*P*-value	*P*-value
Birthday spacings	0.377207	0.640772
Overlapping 5-permutation	0.410588	0.051538
Binary rank (31 × 31)	0.551701	0.900609
Binary rank (32 × 32)	0.940609	0.604265
Binary rank (6 × 8)	0.530332	0.504383
Bit stream	0.428729	0.461876
OPSO	0.493583	0.498226
OQSO	0.582980	0.478843
DNA	0.632916	0.505181
Stream count-the-ones	0.759561	0.853126
Byte count-the-ones	0.605761	0.479987
Parking lot	0.425621	0.412316
Minimum distance	0.522822	0.486276
3D spheres	0.468043	0.414503
Squeeze	0.236035	0.416625
Overlapping sums	0.543661	0.439732
Runs up	0.234988	0.775408
Runs down	0.527703	0.679825
Craps	0.128550	0.423157

**Table 3:** ENT statistical test results for two 80 million bits sequences generated by the proposed Chebyshev polynomial based pseudorandom bit generator and Duffing map based pseudorandom bit generator, respectively.

ENT statistical test	Chebyshev PRBG results	Duffing PRBG results
Entropy	7.999999 bits per byte	7.999999 bits per byte
Optimum compression	OC would reduce the size of this 125000000 byte file by 0%	OC would reduce the size of this 125000000 byte file by 0%
*χ* ^2^ distribution	For 125000000 samples it is 222.98 and randomly would exceed this value 92.68% of the time	For 125000000 samples it is 228.17 and randomly would exceed this value 88.54% of the time
Arithmetic mean value	127.49810 (127.5 = random)	127.5050 (127.5 = random)
Monte Carlo *π* estimation	3.142062386 (error 0.01%)	3.140968178 (error 0.02%)
Serial correlation coefficient	−0.000026 (totally uncorrelated = 0.0)	0.000018 (totally uncorrelated = 0.0)

**Table 4:** Average pixel intensity of plain image colors and encrypted image colors.

File name	Plain image color			Encrypted image color		
	Red	Green	Blue	Red	Green	Blue
4.1.01	75.827	52.559	46.305	127.661	127.203	127.458
4.1.02	42.075	30.086	27.540	128.033	127.233	127.413
4.1.03	137.603	139.958	144.018	127.265	127.394	127.697
4.1.04	129.218	99.267	125.199	127.393	127.275	127.021
4.1.05	146.564	133.000	142.023	127.779	127.268	126.878
4.1.06	132.202	124.902	143.263	127.563	127.377	127.346
4.1.07	179.204	180.650	142.348	127.152	127.325	127.378
4.1.08	174.897	170.866	128.346	126.856	127.426	127.563
4.2.01	176.270	70.494	108.898	127.562	127.118	127.468
4.2.02	234.195	208.644	163.552	127.101	127.729	127.722
4.2.03	137.391	128.859	113.117	127.199	128.126	127.392
4.2.04	180.224	99.051	105.410	127.266	127.311	127.519
4.2.05	177.577	177.852	190.214	127.331	127.565	127.654
4.2.06	131.007	124.304	114.893	127.254	127.850	127.450
4.2.07	149.821	115.568	66.534	127.368	127.379	127.257
House	155.436	168.226	142.209	127.419	127.659	127.525

**Table 5:** Entropy results of plain images and encrypted images.

File name	Plain image color			Encrypted image color		
	Red	Green	Blue	Red	Green	Blue
4.1.01	6.42005	6.44568	6.38071	7.96418	7.96805	7.96648
4.1.02	6.24989	5.96415	5.93092	7.96622	7.96734	7.96629
4.1.03	5.65663	5.37385	5.71166	7.96606	7.96740	7.96398
4.1.04	7.25487	7.27038	6.78250	7.96692	7.96552	7.96741
4.1.05	6.43105	6.53893	6.23204	7.96587	7.96618	7.96776
4.1.06	7.21044	7.41361	6.92074	7.96598	7.96697	7.96786
4.1.07	5.26262	5.69473	6.54641	7.96392	7.96634	7.96632
4.1.08	5.79199	6.21951	6.79864	7.96515	7.96651	7.96782
4.2.01	6.94806	6.88446	6.12645	7.96799	7.96762	7.96848
4.2.02	4.33719	6.66433	6.42881	7.96825	7.96838	7.96582
4.2.03	7.70667	7.47443	7.75222	7.96999	7.96778	7.96859
4.2.04	7.25310	7.59404	6.96843	7.96777	7.96932	7.96998
4.2.05	6.71777	6.79898	6.21377	7.96715	7.96807	7.96883
4.2.06	7.31239	7.64285	7.21364	7.96799	7.96749	7.96791
4.2.07	7.33883	7.49625	7.05831	7.96864	7.96756	7.96730
House	7.41527	7.22948	7.43538	7.96849	7.96825	7.96735

**Table 6:** Horizontal, vertical and diagonal correlation coefficients of adjacent pixels in plain images and encrypted images.

File name	Plain image correlation			Encrypted image correlation		
	Horizontal	Vertical	Diagonal	Horizontal	Vertical	Diagonal
4.1.01	0.956725	0.952503	0.937836	0.001274	0.001785	0.003044
4.1.02	0.908923	0.944135	0.889084	−0.007292	0.007162	0.004493
4.1.03	0.970861	0.916864	0.895799	−0.005882	−0.004236	0.002140
4.1.04	0.956759	0.964448	0.930833	−0.004143	−0.006414	0.008894
4.1.05	0.982138	0.974908	0.962532	0.005939	−0.001269	−0.002035
4.1.06	0.959183	0.934498	0.926566	0.003809	0.011929	−0.002274
4.1.07	0.988603	0.987932	0.979855	−0.008391	0.001379	−0.000308
4.1.08	0.977248	0.979839	0.958275	0.000991	−0.000089	−0.002968
4.2.01	0.978507	0.970863	0.964947	0.001250	−0.000860	0.001454
4.2.02	0.896888	0.909936	0.863983	0.000449	0.001230	−0.000765
4.2.03	0.907119	0.877498	0.839639	−0.001320	−0.000628	−0.000366
4.2.04	0.933223	0.958036	0.918587	−0.004386	0.000342	0.000569
4.2.05	0.962496	0.915378	0.914867	0.004689	0.000547	0.000136
4.2.06	0.969769	0.968659	0.953038	0.000850	0.005358	0.003821
4.2.07	0.964885	0.961169	0.948114	0.001554	−0.001897	0.002504
House	0.975076	0.959036	0.944382	0.001099	−0.002301	0.001799

**Table 7:** NPCR and UACI results of encrypted plain images and encrypted with one pixel difference plane images.

File name	NPCR test			UACI test		
	Red	Green	Blue	Red	Green	Blue
4.1.01	99.5701	99.5911	99.6155	33.6394	33.3493	33.4648
4.1.02	99.6613	99.5743	99.5804	33.4397	33.3669	33.4438
4.1.03	99.6094	99.6216	99.6109	33.3171	33.5476	33.3226
4.1.04	99.6323	99.6384	99.6155	33.4149	33.5338	33.3298
4.1.05	99.5987	99.6201	99.5743	33.4149	33.5670	33.4883
4.1.06	99.5693	99.5972	99.5705	33.4311	33.4601	33.4934
4.1.07	99.5941	99.6094	99.5972	33.5586	33.4576	33.4826
4.1.08	99.6490	99.6414	99.6536	33.4083	33.5591	33.4937
4.2.01	99.6346	99.6033	99.6311	33.4646	33.4548	33.4644
4.2.02	99.6120	99.6056	99.5894	33.4073	33.5304	33.4401
4.2.03	99.6197	99.6021	99.6273	33.4377	33.3873	33.4169
4.2.04	99.6109	99.6094	99.6185	33.4668	33.5337	33.3924
4.2.05	99.6025	99.5975	99.6101	33.4869	33.4080	33.4413
4.2.06	99.6136	99.5953	99.6361	33.3937	33.5238	33.5039
4.2.07	99.6426	99.6231	99.6300	33.4659	33.5129	33.4368
House	99.6357	99.6082	99.6220	33.3519	33.4210	33.4300

**Table 8:** Correlation coefficients between the corresponding pixels of the 48 different encrypted images obtained from the 16 plain images by using the three slightly different secret keys: K1, K2, and K3.

Encrypted image 1	Encrypted image 2	Correlation coefficient	Encrypted image 1	Encrypted image 2	Correlation coefficient
4.1.01.K1	4.1.01.K2	0.006252	4.2.01.K1	4.2.01.K3	0.001802
4.1.02.K1	4.1.02.K2	0.002129	4.2.02.K1	4.2.02.K3	0.000867
4.1.03.K1	4.1.03.K2	0.006434	4.2.03.K1	4.2.03.K3	0.001430
4.1.04.K1	4.1.04.K2	0.001634	4.2.04.K1	4.2.04.K3	0.000064
4.1.05.K1	4.1.05.K2	−0.001745	4.2.05.K1	4.2.05.K3	0.003107
4.1.06.K1	4.1.06.K2	−0.005686	4.2.06.K1	4.2.06.K3	−0.001260
4.1.07.K1	4.1.07.K2	0.000907	4.2.07.K1	4.2.07.K3	−0.001401
4.1.08.K1	4.1.08.K2	−0.003864	House.K1	House.K3	−0.001986
4.2.01.K1	4.2.01.K2	0.000299	4.1.01.K2	4.1.01.K3	−0.002088
4.2.02.K1	4.2.02.K2	0.001053	4.1.02.K2	4.1.02.K3	−0.001454
4.2.03.K1	4.2.03.K2	0.000103	4.1.03.K2	4.1.03.K3	−0.003497
4.2.04.K1	4.2.04.K2	0.001290	4.1.04.K2	4.1.04.K3	−0.002121
4.2.05.K1	4.2.05.K2	0.000557	4.1.05.K2	4.1.05.K3	−0.002167
4.2.06.K1	4.2.06.K2	−0.000290	4.1.06.K2	4.1.06.K3	0.000598
4.2.07.K1	4.2.07.K2	0.001601	4.1.07.K2	4.1.07.K3	0.004454
House.K1	House.K2	0.000905	4.1.08.K2	4.1.08.K3	0.001396
4.1.01.K1	4.1.01.K3	−0.001953	4.2.01.K2	4.2.01.K3	0.004092
4.1.02.K1	4.1.02.K3	0.002054	4.2.02.K2	4.2.02.K3	−0.000099
4.1.03.K1	4.1.03.K3	0.004989	4.2.03.K2	4.2.03.K3	0.000007
4.1.04.K1	4.1.04.K3	0.001796	4.2.04.K2	4.2.04.K3	0.000170
4.1.05.K1	4.1.05.K3	−0.000826	4.2.05.K2	4.2.05.K3	0.002596
4.1.06.K1	4.1.06.K3	0.004114	4.2.06.K2	4.2.06.K3	0.003894
4.1.07.K1	4.1.07.K3	−0.000977	4.2.07.K2	4.2.07.K3	−0.001332
4.1.08.K1	4.1.08.K3	−0.000203	House.K2	House.K3	−0.000282

**Table 9:** Time test (seconds).

Image size	Reference [24]	Reference [32]	Reference [33]	Our scheme
256 × 256	0.22	1.34	0.35	0.19
512 × 512	1.04	5.26	0.72	0.61
